# The impact of *Bacillus subtilis* DSM 32315 on the pathology, performance, and intestinal microbiome of broiler chickens in a necrotic enteritis challenge

**DOI:** 10.3382/ps/pey500

**Published:** 2018-11-18

**Authors:** Rose A Whelan, Kiran Doranalli, Teemu Rinttilä, Kirsi Vienola, German Jurgens, Juha Apajalahti

**Affiliations:** 1 Evonik Nutrition & Care GmbH, Hanau, 63067, Hessen, Germany; 2 Alimetrics Ltd, Espoo, 02920, Finland

**Keywords:** broiler, necrotic enteritis, *Bacillus subtilis*, probiotic, microbiome

## Abstract

It was hypothesized that dietary inclusion of *Bacillus subtilis* DSM 32315 could inhibit *Clostridium perfringens* induced necrotic enteritis (NE), thereby improving broiler performance. Male, d 0 chicks were randomly assigned 14 birds/pen, 11 pens/treatment in 3 treatments: a basal diet (control), a coccidiostat fed control (Narasin), and a direct fed microbial (DFM) *B. subtilis* DSM 32315 treatment. Necrotic enteritis was induced in all birds by oral inoculation of *Eimeria maxima* oocysts on d 12 and a virulent *C. perfringens* on d 16. Mortality was reduced (*P <* 0.001) in DFM and Narasin compared to control. DFM reduced (*P <* 0.001) feed conversion ratio (FCR) compared to control. Furthermore, DFM and Narasin reduced (*P <* 0.001) footpad lesions. The DFM was shown to increase (*P <* 0.05) *Bacillus* spp. and decrease (*P <* 0.05) *C. perfringens* in the ileum and cecum at several time points. To investigate microbiome changes in the cecum, digesta samples were analyzed with % guanine and cytosine (%G+C) microbial profiling which fractionates bacterial chromosomes based on the %G+C in DNA. The method revealed treatment profile peaks in low (27.0 to 34.5%), mid (40.5 to 54.0%), and high (59.0 to 68.0%) G+C fractions. 16S rRNA gene amplification and high throughput sequencing was conducted on each of these fractions in order to elucidate specific bacterial population differences. In the low and mid %G+C fractions, DFM had greater abundance of *Lactobacillaceae* family members (*P* = 0.03 and *P* = 0.01, respectively) and *Lactobacillus salivarius* (*P* = 0.04 and *P* = 0.01, respectively) than control or Narasin. *Lactobacillus johnsonii* was also greater in the low %G+C fraction compared to control and Narasin (*P* = 0.01). *Lachnospiraceae* (*P* = 0.04) and *Ruminococcaceae* (*P* < 0.01) in the mid %G+C fraction were reduced in the DFM compared to control. Positive alterations to the microbial populations in the gut of broilers may at least be a partial mechanism by which *B. subtilis* DSM 32315 reduced pathology and improved performance of broilers in the NE challenge.

## INTRODUCTION

Poultry production has become increasingly more efficient in the last 50 years due to significant advancements in genetics, nutrition, and management. It has been reported that since the 1950’s growth rates of broilers increased by more than 400%, feed conversion ratio (**FCR**) was reduced by around 50%, and body weight (**BW**) that once required over 100 d of growth can now be attained in 35 d (Havenstein et al., 2003; Zuidhof et al., 2014). However, in order to achieve the high genetic potential for growth performance, broiler flocks must receive not only ideal environmental conditions and nutrition, but must also be free from health challenges. Intestinal pathogenic diseases in poultry can be especially detrimental to efficient growth for a multitude of reasons, including damage to the intestines that impairs digestion and uptake of nutrients, a decrease in feed intake as a physiological response to the infection, and a reduction in anabolic muscle deposition or even muscle catabolism to support the nutrient requirements of an activated immune system (Remus et al., 2014). The ubiquitous poultry disease, known as necrotic enteritis (**NE**), is one such example of an intestinal disease with major impacts on performance. A recent meta-analysis study reported that an affliction with *Clostridium* spp. disease in broiler chickens reduces average daily feed intake by 40% and average daily gain by 16% (Remus et al., 2014). These NE-related performance losses have been estimated to cost between US$878.19 and 1,480.52 per flock of 20,000 birds in mild disease with low mortality (Skinner et al., 2010). Taking this into account with more severe forms of the disease resulting in increased mortality, veterinary intervention, and therapeutic treatment costs, NE is assumed to have major global impacts on the cost efficiency of broiler meat production.

Many intestinal diseases, like NE, are the result of a dysbiosis in the intestinal microbiome that leads to the outgrowth and virulence gene expression of opportunistic pathogens. Necrotic enteritis occurs when resident populations of the bacterial species *Clostridium perfringens* are able to proliferate and produce toxins, which results in necrotizing lesions most commonly occurring in the ileum (Timbermont et al., 2011; Prescott et al., 2016). Antibiotics can be used to inhibit the growth and subsequent toxin gene expression in *C. perfringens* to prevent the occurrence of NE (Lanckriet et al., 2010). However, the prophylactic use of antibiotics is banned in many regions like the European Union and is being phased out of production due to consumer demand in others over the risk of increasing antibiotic resistance in pathogens of veterinary and human medical relevance (Lekshmi et al., 2017). Alternative strategies are, therefore, being investigated to control intestinal pathogenic disease in poultry (Dahiya et al., 2006; Stringfellow et al., 2009; Timbermont et al., 2010; M'Sadeq et al., 2015). Probiotics, also known as direct fed microbials (**DFM**), are of particular interest as feed additives in the control of intestinal disease due to the many potential modes of action a strain may exhibit to control populations of disease causing organisms in the gut. For example, live probiotic bacteria have been observed to support physiological and immune development of the host for improved disease resistance, compete with pathogens for nutrients and adhesions, produce metabolites that can directly inhibit the growth of disease causing bacteria, and support intestinal colonization of other potentially beneficial microbial populations (Ducatelle et al., 2015; Elshaghabee et al., 2017).

For the poultry industry, *Bacillus* spp. have been highly interesting as in-feed probiotics due to their ability to produce spores which can be resistant to the high heat utilized in modern production of pelleted poultry feeds, and can additionally be tolerant to the low pH, bile, and enzymes encountered in the upper gastrointestinal (**GI**) tract of the chicken (Elshaghabee et al., 2017). Once in the intestines of the bird, the transient *Bacillus* spores can germinate and produce secondary metabolites with potential health benefits for the host animal. The *Bacillus subtilis* strain DSM 32315 was selected based on heat stability, pH and bile resistance, and metabolite expression for use in poultry feeds. Due to its ability to inhibit the growth of *C. perfringens* in vitro, it is hypothesized to be an effective feed additive for the control of NE in poultry (unpublished data).

The present study was conducted in order to investigate whether inclusion of *B. subtilis* DSM 32315 spores in poultry feed could prevent the pathology and associated performance detriments associated with NE in a challenge trial in a comparable way to an antimicrobial agent. The potential effects of in-feed *B. subtilis* DSM 32315 spores on the intestinal microbiome were additionally examined to investigate whether the DFM exhibits a microbiome-stabilization mode of action that could prevent enteric disease of poultry resulting from microbiome dysbiosis.

## MATERIALS AND METHODS

### Animals and Diets

All live animal research met the guidelines approved by the institutional animal care and use committee. Four-hundred-and-sixty-two-day-old male chicks (Ross 308) of an average initial BW of 47.2 + 0.1 g were randomly assigned to 3 dietary treatments with 11 replicate pens of 14 birds each, with pen as the experimental unit. Pens were 1.125 m^2^ with wood shavings/chopped straw litter. The temperature of the hall was 32°C upon chick arrival with brooder lamps to provide extra heating in the first week. The temperature was gradually decreased to 22°C over the rearing period. Luminosity was adjusted to 20 lux and dark hours were gradually increased within a week to 18 h light and 6 h dark daily.

Birds had ad libitum access to feed and water. Corn-soybean meal based basal diets were formulated to meet Evonik amino acids recommendations (AMINOChick® 2.0, Hanau, Germany) for starter (d 0 to 11), grower (d 12 to 25), and finisher (d 26 to 35) phases (Table 1). The starter diet was prepared as 2 mm crumbled pellets and the grower and finisher diets as 4 mm pellets. Dietary treatments included a basal diet (control), a basal diet supplemented with 65 g/MT of Narasin (hereafter referred as narasin), and a basal diet supplemented with 500 g/MT of the product GutCare® PY1 (Evonik Nutrition & Care GmbH., Hanau, Germany) containing 2 × 10^9^ cfu/g of *B. subtilis* DSM 32315 spores (DFM).

**Table 1.** Ingredient and nutrient composition of basal diet (%, as-fed unless otherwise noted).

Item	Starter (d 0 to 11)	Grower (d 12 to 25)	Finisher (d 26 to 35)
Corn	52.17	57.10	61.94
Soybean meal	40.06	35.45	30.33
Soybean oil	3.28	3.68	4.12
Monocalcium phosphate	1.76	1.47	1.31
Mineral premix^1^	0.20	0.20	0.20
Vitamin premix^2^	0.20	0.20	0.20
Caclium carbonate	1.40	1.18	1.11
Sodium chloride	0.37	0.37	0.37
Choline chloride (60%)	0.09	0.09	0.09
DL-Methionine	0.30	0.23	0.23
L-Lysine–HCl	0.13	0.03	0.08
L-Threonine	0.04	0.00	0.02
Calculated composition			
ME^3^, kcal/kg	2,950	3,025	3,100
CP^4^	22.96	21.50	19.50
SID^5^ lys	1.23	1.08	1.00
SID met + cys	0.88	0.80	0.76
SID thr	0.79	0.70	0.65
SID val	0.95	0.89	0.81
SID arg	1.44	1.35	1.20

^1^Contents of the mineral premix: calcium 296.9 g/kg, zinc 32.5 g/kg, manganese 25.0 g/kg, iron 12.5 g/kg, copper 4.0 g/kg, iodine 225 mg/kg, and selenium 100 mg/kg.

^2^Contents of the vitamin premix: calcium 331.3 g/kg, all-rac-α-tocopheryl acetate 30.0 g/kg, niacin 20.1 g/kg, pantothenic acid 7.51 g/kg, riboflavin 3.0 g/kg, pyridoxine 2.01 g/kg, retinol 1.8 g/kg, menadione 1,505 mg/kg, thiamine 1,257 mg/kg, folic acid 504 mg/kg, biotin 75.0 mg/kg, cholecalciferol 56.3 mg/kg, and cobalamin 12.5 mg/kg.

^3^ME = metabolizable energy.

^4^CP = crude protein.

^5^SID = standard ileal digestibility.

### Necrotic Enteritis Challenge

To induce an NE challenge, all birds were orally inoculated on d 12 with 5,000 *Eimeria maxima* oocysts and on d 16 with between 0.5 and 1 × 10^9^ live cells of a pathogenic field strain of *C. perfringens* isolated from the ileum of broilers diagnosed with NE.

### Physiological Outcomes

Body weight and feed intake were recorded on a pen basis on d 35, then calculated and reported as averages per bird. Feed conversion ratio and mortality adjusted FCR (**aFCR**) were calculated on a pen basis for the period between d 1 and 35. Mortalities were calculated in the post-challenge period from d 12 to 35 and were reported as a percentage of pens and reported as an average of all pen replicates in a treatment. Footpad lesions were scored on d 35 utilizing a 0 to 4 scale: 0 (representing no evidence of pododermatitis) to 4 (severe pododermatitis) based on Welfare Quality 2009 (Rushen et al., 2011).

### Targeted Bacteria Enumeration in Digesta

Digesta from ileum and cecum was collected from 1 bird/pen from each of the 11 replicate pens/treatment on d 11, 18, and 35. Removal of undigested particles from the digesta samples was performed by washing the samples with repeated low-speed centrifugation, followed by collection of the bacteria in the pooled supernatants with high-speed centrifugation (Apajalahti et al., 1998). Cell lysis and DNA extraction from ileal and cecal bacterial pellets were performed as described by Apajalahti et al. (1998).

Quantitative real-time PCR (**qPCR**) analysis was conducted to quantify the abundance of *B. subtilis* group and *C. perfringens* by enumerating the target gene copies (16S rRNA for *B. subtilis* group and phospholipase C (*plc*) for *C. perfringens*) per gram of digesta (Tansuphasiri et al., 2002). The target gene was the phospholipase c (*plc*) for *C. perfringens* and 16S rRNA *B. subtilis* group. The annealing temperatures and target gene sequences are given in Table 2.

**Table 2.** Target microorganisms and genes, annealing temperatures, and primer sequences used in the quantitative real-time PCR (qPCR) analysis of samples from the necrotic enteritis (NE) challenge trial with broiler chickens.

Target group or microorganism	Annealing temperature (°C)	Target gene	Primer sequence (5′–3′)	Reference
*Bacillus subtilis* group	63	16S rRNA	F: TTGATCTTAGTTGCCAGCATTC R:ACAGATTTGTGGGATTGGCTTA	Designed for the present study
*Clostridium perfringens*	62	*plc*	F: TTACCTTTGCTGCATAATCCC R:ATAGATACTCCATATCATCCTGCT	Tansuphasiri et al. (2002)

The qPCR analysis was performed with an ABI Prism Sequence Detection System 7500 instrument (Life Technologies, Frederick, MD). The amplifications were performed in a volume of 15 μl with SYBR Select Master Mix (Life Technologies, Frederick, MD), 0.25 μM of both primers, and 5 μl of 1:100 (ileal samples) or 1:1,000 (cecal samples) diluted template DNA or deionized sterile water as a no-template control. The thermal cycling involved 1 cycle of preheating (50°C for 2 min), initial denaturation step (95°C for 10 min), 40 cycles of denaturation (95°C for 15 s), and primer annealing and extension (primer-specific annealing temperature for 60 s). To determine the specificity of the PCR reactions, a melting curve analysis was carried out in conjunction with each amplification run by slow cooling from 95°C to 60°C, with fluorescence collection at 0.3°C intervals.

Tenfold serial dilutions ranging from 1 × 10^8^ to 1 × 10^2^ copies of synthetic standard target gene was included on each 96-well plate. The fractional cycle number at which the fluorescence passes the threshold (set at 0.3 fluorescent units) was determined for the unknowns and compared with the standard curves. Taking into account the original mass of starting material, elution volume, and PCR template dilution, the number of target gene copies was determined per gram (wet weight) of intestinal material. Finally, target gene copy numbers were log_10_ transformed to achieve a normal distribution, and the average as well as standard error of the mean from microbial results was calculated for each treatment, respectively.

### Cecal Microbiome Determination

#### %G+C Profiling.

Cecal digesta samples from 2 birds/pen of each of the 11 replicate pens/treatment were collected on d 18 and bacterial DNA was extracted as previously described (Apajalahti et al., 1998). The DNA samples were then fractionated by 72 h CsCl equilibrium density gradient ultracentrifugation (100,000× g), which separates chromosomes with different guanine and cytosine (**G**+**C**) content (Apajalahti et al., 1998). This separation is based on differential density imposed by the adenine–thymine-dependent DNA-binding dye bisbenzimidazole. Following the ultracentrifugation, the formed gradients were pumped through a flow-through UV absorbance detector set to 280 nm. Finally, the %G+C content represented by each gradient fraction was determined by linear regression analysis (*r*^2^ > 0.99).

#### 16S Gene Sequencing of Select %G+C Profile Fractions.

A second round of %G+C profiling was conducted on 5 replicate DNA samples/treatment selected for sequencing. During the ultracentrifugation step (previously described), the formed gradients were pumped through a flow-through UV absorbance detector set to 280 nm and %G+C fractions were collected at 5 to 7% intervals. The collected DNA fractions were subjected to desalting with PD-10 columns (GE Healthcare, Liittle Chalfont, UK) for subsequent 16S rRNA gene PCR amplification with the universal broad-range primer pair. The PCR products were sequenced with the Illumina MiSeq (Illumina, San Diego, CA) sequencing platform. Sequence reads were submitted to the European Nucleotide Archive (https://www.ebi.ac.uk/ena) under accession numbers ERS2265158 and ERS2265198. Direct URL to access the study is: http://www.ebi.ac.uk/ena/data/view/PRJEB25328. Raw sequence data was subjected to standard sequencing data pre-processing and data analysis: demultiplexing of all libraries for each sequence lane using Illumina bcl2fastq 1.8.4. software (Illumina, San Diego, CA), sorting of reads by amplicon inline barcodes, clipping of the adapters, primer-based sorting, sequence alignment, combining of the forward and reverse reads using BBMerge 34.48, generating consensus sequences, and grouping. 16S rRNA gene data was processed and operational taxonomic units (**OTU**) were picked from amplicons with Mothur 1.35.1 program: alignment was done against 16S rRNA SILVA SEED r119 reference alignment; short alignments (truncated or unspecific PCR products) and chimeras were filtered; sequences were taxonomically classified against the SILVA databases and sequences from other domains of life were removed; OTU were picked by clustering at the 97% identity level; and OTU consensuses were taxonomically classified to genus level.

Operational taxonomic unit diversity analysis was performed with QIIME 1.9.0 (Caporaso et al., 2010): within-sample diversity was analyzed at minimum and median sample sequence count rarefaction levels (“alpha diversity”), including creation of plots and tables with taxonomical sample composition; between-sample diversity was analyzed at minimum and median sample sequence count rarefaction levels (“beta diversity”).

### Statistics

Statistics were analyzed in SAS software v9.4 (SAS Institute Inc., Heidelberg, Germany). The procedure PROC GLM was used for one-way ANOVA with least square difference means as a post-hoc test for treatment comparison of BW, feed intake, FCR, aFCR, mortality, and percentage of microbial family and species population abundance. Percentage data underwent arcsine transformation prior to ANOVA analysis. The PROC NPAR1WAY procedure was used to compare footpad lesions between treatments with the Kruskal-Wallis test of the Wilcoxon rank sums. Significance was reported for all analyses for *P* < 0.05.

## RESULTS

### Broiler Performance, Mortality and Footpad Lesions in the Necrotic Enteritis Challenge

The growth performance variables, mortality, and footpad lesion scores are given in Table 3. The 35 d BW of broilers was unaffected by treatment (*P* = 0.244). Feed intake of birds fed the Narasin treated diet was reduced by 285.9 g (6.9%) compared to the basal diet fed controls (*P* < 0.001), but was unaffected by the DFM treatment compared to the controls. The FCR and aFCR were both lowest in the Narasin treatment group, followed by the DFM treated group and highest in the controls (*P* < 0.001). The post-challenge mortality per pen was 9.1% and 6.5% lower (*P* < 0.001) in the Narasin and DFM treatment groups, respectively, compared to the controls. There was a significant effect of dietary treatment on footpad lesion score based on a rank order comparison (*P* < 0.001) with the controls having the highest mean score (2.29), followed by the DFM (1.77) and then Narasin (0.41) treatments.

**Table 3.** Growth performance, mortality, and footpad lesion scores of broilers in an induced necrotic enteritis (NE) challenge fed from a basal diet, an antibiotic supplemented diet or a direct fed microbial supplemented diet.^1,2,3,4,5^

Treatment	Weight (g/bird)	Feed intake (g/bird)	FCR (g/g)	aFCR (g/g)	Mortality (% of pen)	Footpad lesions (score 0 to 4)
Control	2802.0	4145.8^A^	1.605^A^	1.505^A^	10.4^A^	2.29
Narasin	2778.4	3859.9^B^	1.431^C^	1.425^C^	1.3^B^	0.41
DFM	2845.5	4071.7^A^	1.493^B^	1.456^B^	3.9^B^	1.77
SEM	88.9	114.2	0.069	0.024	4.3	N.a.
*P*-value^1^	0.244	<0.001	<0.001	<0.001	<0.001	<0.001

^1^Results are reported as means of 11 replicate pens.

^2^Weight, feed intake, feed conversion ratio (FCR), and mortality adjusted FCR (aFCR) are reported for the period of d 0 to 35 and statistically analyzed with ANOVA and post-hoc least square difference test.

^3^Mortality percentages were reported for d 12 to 35 and converted with arcsin prior to one-way ANOVA analysis.

^4^Footpad lesions were reported on d 35 on a 0 to 4 scale and analyzed with Kruskal-Wallis test.

^5^Means with different superscripts are significantly different (*P* < 0.05).

### Targeted Bacteria Enumeration in Digesta of Broilers in the Necrotic Enteritis Challenge

The quantification of *B. subtilis* group specific 16S rRNA gene copies in the ileum and cecum digesta of broilers at d 11, 18, and 35 is given in Table 4. Compared to controls, the DFM fed treatment group had consistently higher *B. subtilis* in the ileum (log_10_ gene copies/g digesta) at d 11, 18, and 35 by 1.45 (*P* < 0.001), 0.77 (*P* = 0.036), and 1.34 (*P* < 0.001), respectively. At d 11, the ileum *B. subtilis* 16S rRNA gene copy numbers were also higher in the Narasin treatment group compared to the controls (*P* < 0.001), while at d 18 and 35 *B. subtilis* quantified in the ileum of the Narasin group did not differ from the controls. In the cecum digesta, the DFM fed treatment group had consistently higher *B. subtilis* (log_10_ gene copies/g digesta) compared to the controls at d 11, 18, and 35 by 2.41(*P* < 0.001), 1.00 (*P* = 0.002), and 1.33 (*P* < 0.001), respectively. Similar to the ileum findings, the Narasin treatment group increased *B. subtilis* (log_10_ gene copies/g digesta) enumerated in the cecum at d 11 by 1.14 (*P* < 0.001) compared to the controls, but was statistically similar to controls at d 18 and 35.

**Table 4.** Enumeration of *Bacillus subtilis* group (16S rRNA gene copies/g digesta) in ileum and cecum digesta from broilers in an induced necrotic enteritis (NE) challenge fed a basal diet, an antibiotic supplemented diet, or a direct fed microbial supplemented diet with quantitative real-time PCR (qPCR) at d 11, 18, and 35.^1,2,3^

	Ileum (log_10_ gene copies/g digesta)			Cecum (log_10_ gene copies/g digesta)		
Treatment	D 11	D 18	D 35	D 11	D 18	D 35
Control	4.75^C^	4.71^B^	4.77^B^	4.10^C^	4.58^B^	5.06^B^
Narasin	5.68^B^	4.94^AB^	4.84^B^	5.24^B^	4.54^B^	5.23^B^
DFM	6.20^A^	5.48^A^	6.11^A^	6.51^A^	5.58^A^	6.39^A^
SEM	0.72	0.99	0.32	0.80	1.05	0.79
*P*-value^1^	<0.001	0.036	<0.001	<0.001	0.002	<0.001

^1^Results are reported as means of 1 representative bird/pen of 11 replicate pens/treatment.

^2^Statistical analyses were conducted by ANOVA and post-hoc least square difference test.

^3^Means with different superscripts are significantly different (*P* < 0.05).

The quantification of *C. perfringens* specific *plc* gene copies in the ileum and cecum digesta of broilers at d 11, 18, and 35 is given in Table 5. At d 11, prior to the induction of the NE challenge with either *Eimeria* spp. or *C. perfringens* inoculation, there was a trend (*P* = 0.062) towards a reduction in *C. perfringens* quantified in the cecum digesta of DFM fed birds compared to controls and Narasin fed birds. Dietary treatment was observed to affect *C. perfringens* populations at both time points after induction of NE and in both ileum and cecum digesta (*P* < 0.001 for both time points and intestinal sections). In the ileum, the Narasin treatment group had the lowest levels of *C. perfringens* (log_10_ gene copies/g digesta), with a reduction of 1.67 (*P* < 0.001) at d 18 and 1.33 (*P* < 0.001) at d 35 compared to controls. The *C. perfringens* populations in the ileum of DFM fed birds were significantly higher than those in the Narasin treatment group at both d 18 (*P* < 0.001) and 35 (*P* < 0.001). However, compared to the controls, the DFM treatment did reduce *C. perfringens* in the ileum at d 18 and 35, by 0.69 and 0.63 (log_10_ gene copies/g digesta), respectively. In cecum samples at d 18 and 35, the DFM and control group had statistically similar levels of *C. perfringens*. In the cecum at d 18, Narasin fed birds were observed to have 1.02 (*P* < 0.001) lower *C. perfringens* (log_10_ gene copies/g digesta) than the DFM fed birds and 1.27 (*P* < 0.001) lower *C. perfringens* (log_10_ gene copies/g digesta) than controls. Similarly, Narasin fed birds were observed to have lower *C. perfringens* (log_10_ gene copies/g digesta) in the cecum at d 35 than the DFM by 1.14 (*P* < 0.001) and the control group by 1.83 (*P* < 0.001).

**Table 5.** Enumeration of *Clostridium perfringens* (*plc* gene copies/g digesta) in ileum and cecum digesta from broilers in an induced necrotic enteritis (NE) challenge fed a basal diet, an antibiotic supplemented diet, or a direct fed microbial supplemented diet with quantitative real-time PCR (qPCR) at d 11, 18, and 35.^1,2,3^

	Ileum (log_10_ gene copies/g digesta)			Cecum (log_10_ gene copies/g digesta)		
Treatment	D 11	D 18	D 35	D 11	D 18	D 35
Control	4.51	6.27^A^	5.36^A^	6.39^A^	6.14^A^	6.64^A^
Narasin	4.18	4.60^C^	4.03^C^	6.38^A^	4.87^B^	4.81^B^
DFM	4.26	5.58^B^	4.73^B^	6.06^B^	5.89^A^	5.96^A^
SEM	0.51	1.07	0.99	0.50	1.10	1.26
*P*-value^1^	0.096	<0.001	<0.001	0.062	<0.001	<0.001

^1^Results are reported as means of 1 representative bird/pen of 11 replicate pens/treatment.

^2^Statistical analyses were conducted by ANOVA and post-hoc least square difference test.

^3^Means with different superscripts are significantly different (*P* < 0.05).

### %G+C Profiling and Sequencing

**Figure 1.**
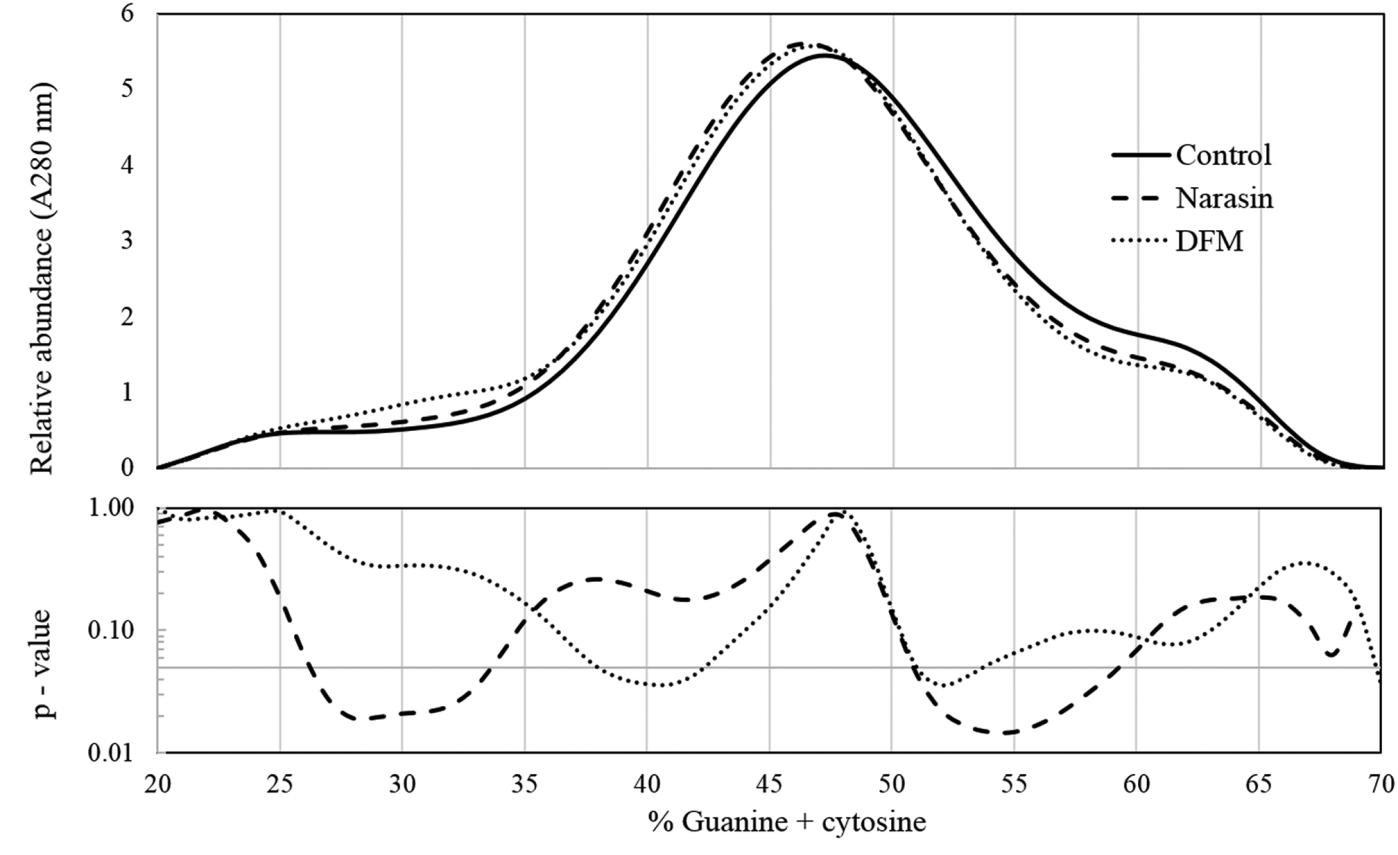
The top graph shows the average % guanine + cytosine (%G+C) profile of microbial DNA extracted from the cecal digesta of 18-day-old broilers fed a control diet, or diets, including Narasin or a direct fed microbial. The lower portion of the graph shows the *P*-value when the average relative abundance of microbial genomes at a specified %G+C were compared with student's *t* tests between either Narasin or direct fed microbial treatments and the control. The horizontal marker indicates the *P* = 0.05, anything below this line is considered significant.

Isolated bacterial DNA from cecal samples obtained during the trial from each of the three treatments was used for %G + C profiling and sequencing of 3 valid fractions where apparent differences in curves was observed: low (27.0 to 34.5%), mid (40.5 to 54.0%), and high (59.0 to 68.9%) fractions (Figure 1). High throughput sequencing of the 16S rRNA genes amplified from these 3 regions was then conducted.

#### High %G+C Profile Sequencing.

**Figure 2.**
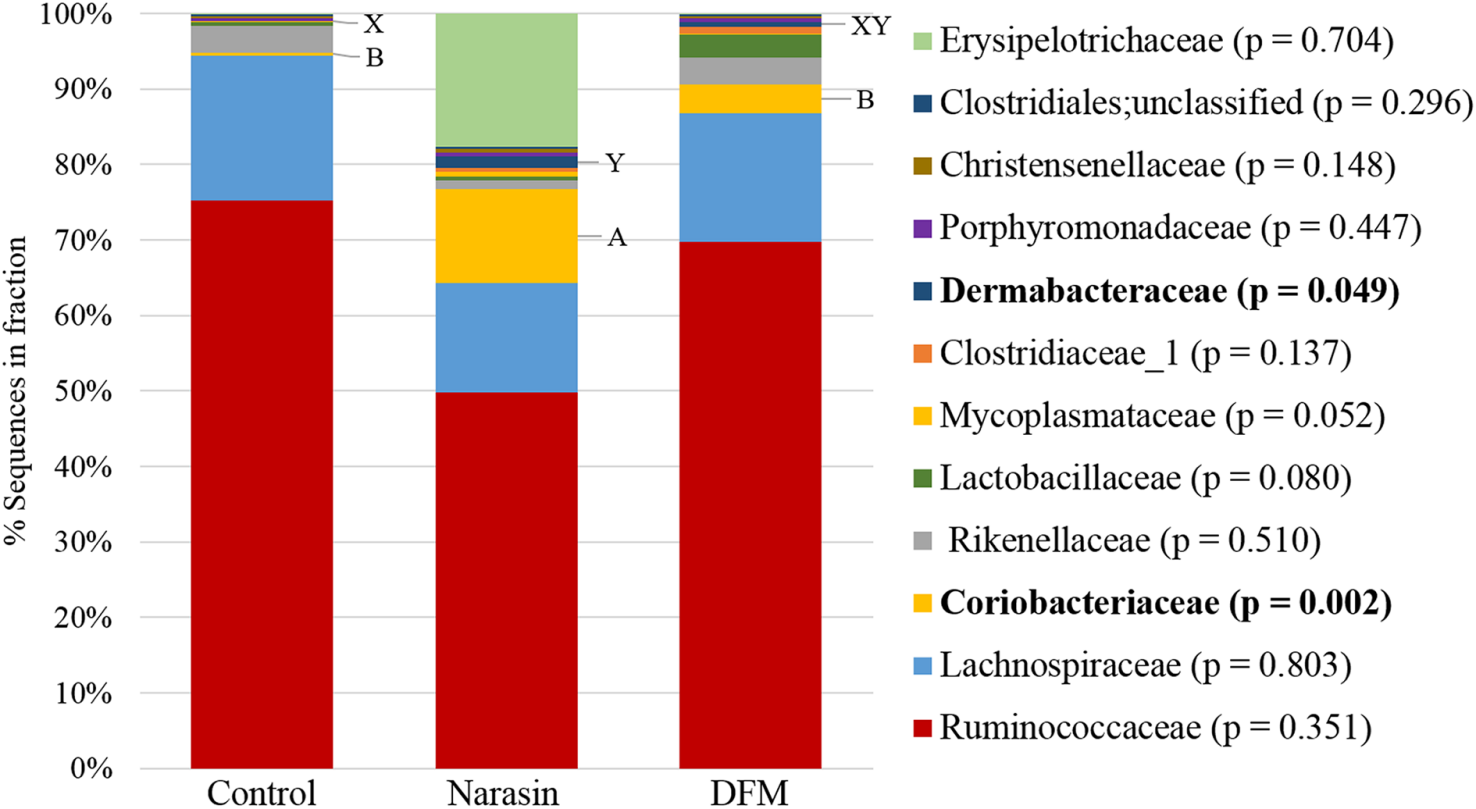
The average relative abundance of the phylogenetic bacterial families identified in the high fraction (59.0 to 68.9%) of the % guanine and cytosine (%G+C) profiles from the cecal microbiome of the control, Narasin, and direct fed microbial treatment groups.

In the high %G+C fraction (Figure 2), Firmicutes were the most abundant phylum represented with members of *Ruminococcaceae* and *Lachnospiraceae* families comprising the majority of sequences in all treatments. However, there were no significant treatment effects observed on the abundance of any families within the Firmicutes phylum. There were treatment effects observed for the quantities of various members of the phylum Actinobacteria. *Coriobacteriaceae* members were represented as a higher (*P* = 0.002) percentage of the bacteria in the ceca of Narasin (13.7%) fed birds compared to control (0.5%) or DFM fed birds (3.8%). Of the OTU that could be classified on a species level, *Eggerthella lenta* was the only identified species in the *Coriobacteriaceae* family. However, there were no significant differences between treatments in the abundance of *E. lenta* (*P* = 0.482). The Narasin treatment also increased the percentage of *Dermabacteraceae* (1.8%) in the cecum bacteria populations compared to control (0.02%), but not in the DFM treatment (0.7%). None of the OTU that could be classified on a species level was members of this family.

#### Mid %G+C Profile Sequencing.

**Figure 3.**
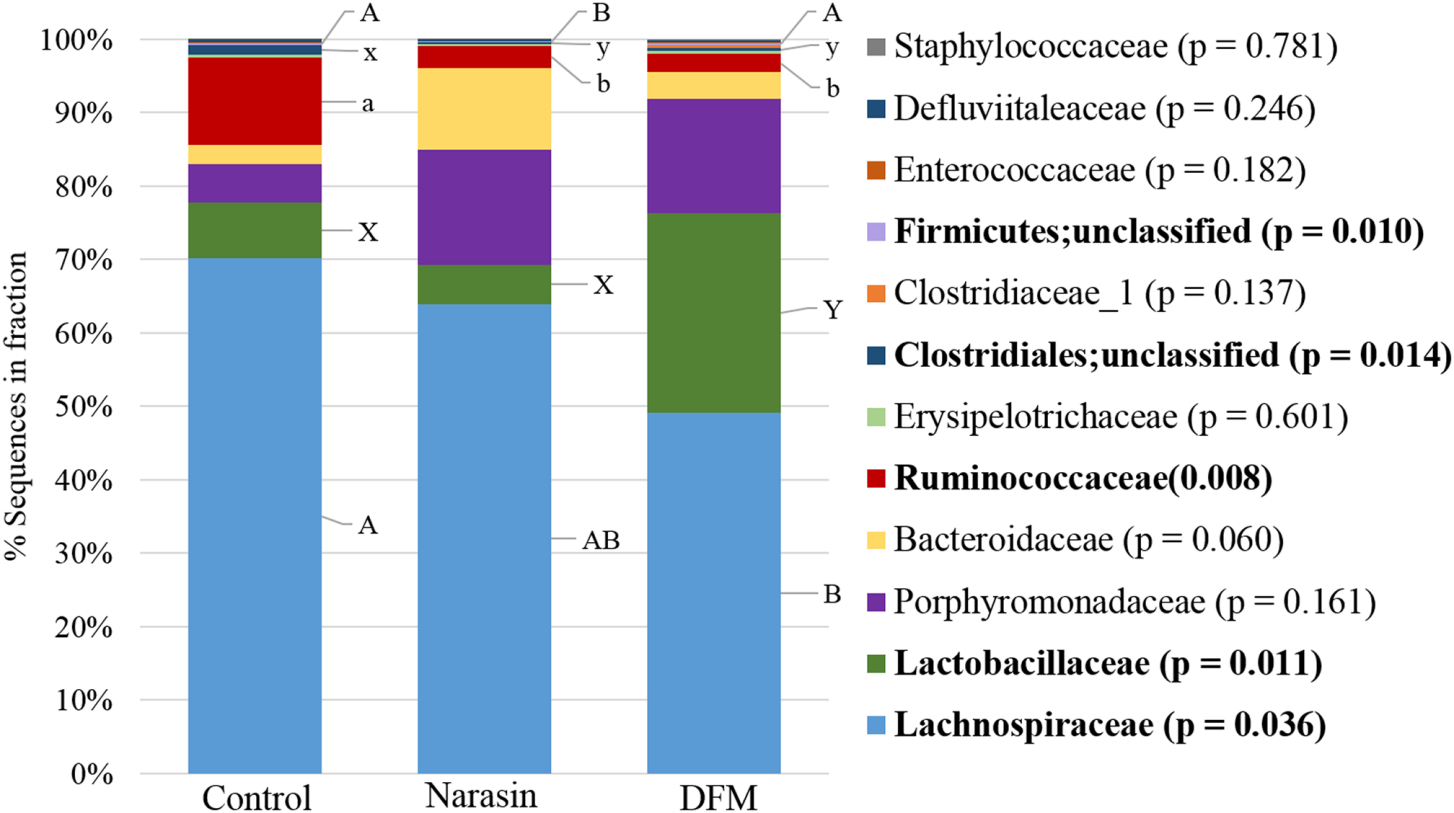
The average relative abundance of the phylogenetic bacterial families identified in the mid fraction (40.5 to 54.0%) of the % guanine and cytosine (%G+C) profiles from the cecal microbiome of the control, Narasin, and direct fed microbial treatment groups.

**Figure 4.**
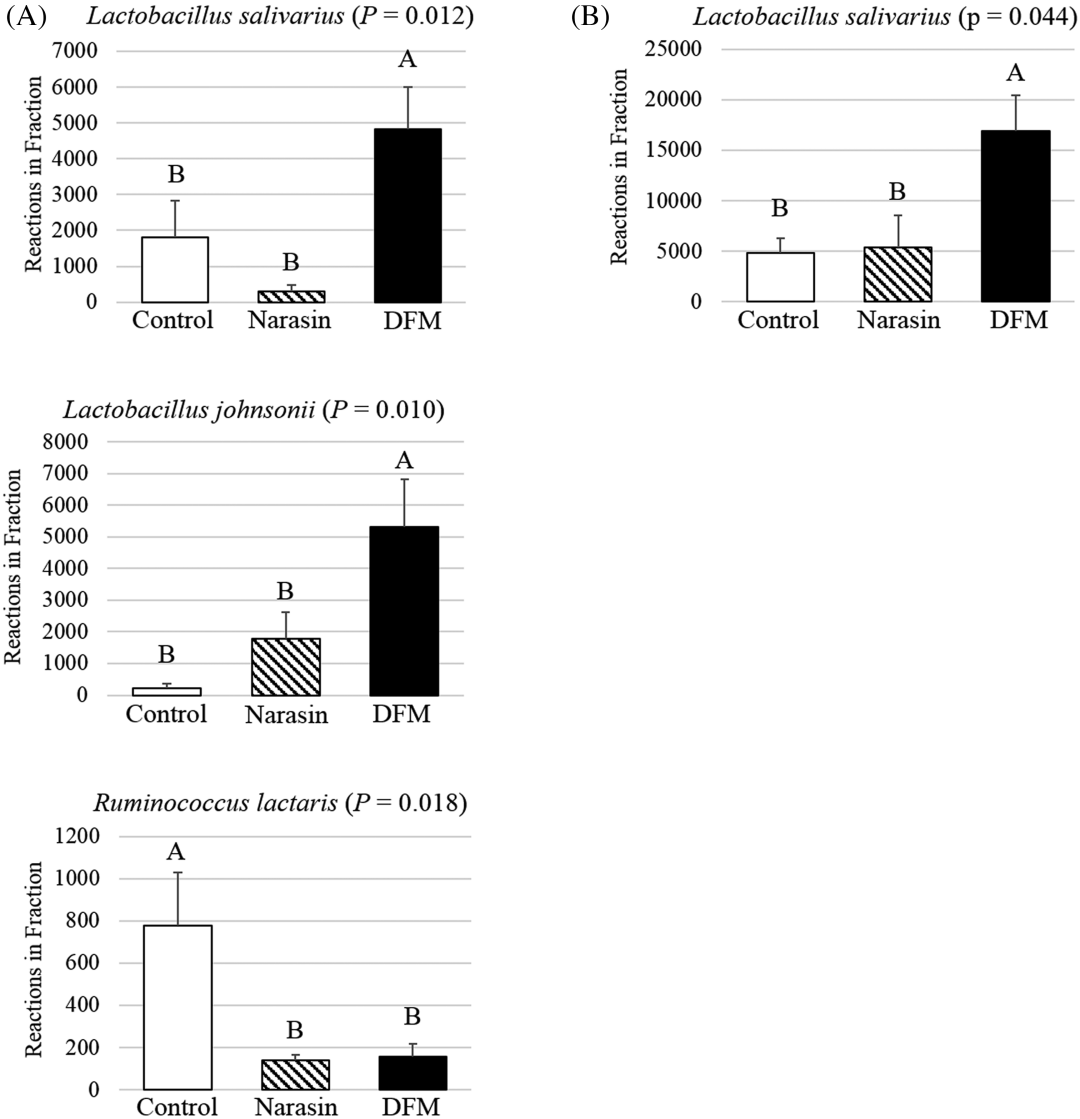
The average relative abundance of 16S rRNA gene operational taxonomic units (OTU) affiliated with different phylogenetic bacterial species sequenced from the low (A) and mid (B) % guanine and cytosine (%G+C) fraction of samples from the control, Narasin, and DFM dietary treatment groups. Statistical analyses were conducted by ANOVA and post-hoc least square difference test. Means with different superscripts are significantly different (*P* < 0.05).

In the mid %G+C fraction (Figure 3), the significant cecal microbiome changes identified between treatment groups were all families belonging to the phylum Firmicutes and this was also the most abundant phylum represented. The *Lachnospiraceae* family was the most prevalent in all treatment groups in this fraction. There were fewer (*P* = 0.036) *Lachnospiraceae* members in the ceca of DFM birds (48.9%) compared to the control (68.9%), while the Narasin treatment (63.6%) did not significantly alter the abundance of this family compared to either the probiotic or control groups. Within this family, there was also a significant decrease (*P* = 0.018) in *Ruminococcus lactaris* sequences identified in the DFM samples compared to both the control and Narasin treatments (Figure 4A, *P* = 0.018). The abundance of *Ruminococcaceae* was significantly reduced (*P* = 0.008) in the bacterial populations from the DFM (2.5%) and Narasin (3.1%) treatments compared to the control (11.6%). A small proportion of OTU from the cecal bacterial chromosomes in the mid %G+C fraction of all treatments was not classifiable on a family level; however, significant effects of treatment were observed in some of these populations. The abundance of unclassified family members belonging to the Clostridiales order was significantly reduced in the DFM (0.4%) and Narasin (0.3%) treatment groups compared to the control (1.3%). Additionally, OTU comprising unclassified family members in the Firmicutes phylum were reduced in the Narasin (0.1%) treatment group compared to the DFM (0.3%) or control groups (0.2%). The abundance of OTU belonging to the *Lactobacilliaceae* family was increased (*P* = 0.011) in the cecal microbiome of DFM (27.0%) treated broilers compared to the control (7.4%) and Narasin (5.3%) groups. Within the *Lactobacilliaceae* family, both *Lactobacillus salivarius* (Figure 4A, *P* = 0.012) and *Lactobacillus johnsonii* (Figure 4A, *P* = 0.010) were more abundant in the DFM treatment than the control or Narasin treatments.

#### Low %G+C Profile Sequencing.

**Figure 5.**
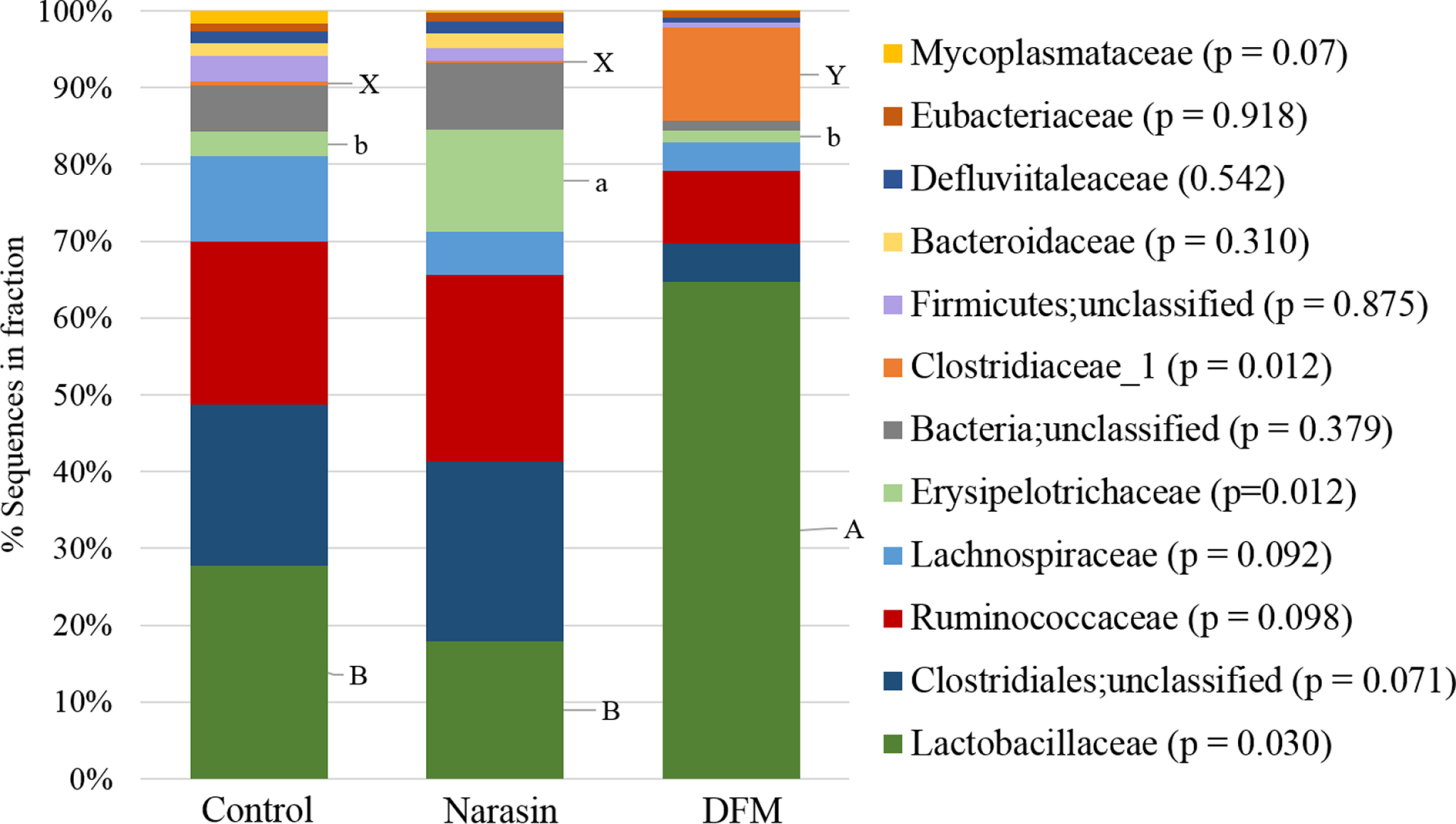
The average relative abundance of the phylogenetic bacterial families identified in the low fraction (27.0 to 34.5%) of the % guanine and cytosine (%G+C) profiles from the cecal microbiome of the control, Narasin, and direct fed microbial treatment groups.

In the low %G+C fraction, the majority of bacterial chromosomes represented belonged to the Firmicutes phylum (Figure 5). In the control and DFM treatments, *Lactobacillaceae* was the largest family represented in the bacterial OTU identified. The abundance of OTU in the *Lactobacillaceae* family was also significantly higher (*P* = 0.030) in DFM treatment (63.7%) compared to the negative control (26.6%) or Narasin group (17.0%). The most abundant species identified in the low %G+C fraction from all treatment groups was a member of the *Lactobacillaceae* family, *L. salivarius*. There were also more (*P* = 0.044) *L. salivarius* identified in this fraction from ceca of DFM treated birds compared to the controls or Narasin treatment group (Figure 4B, *P* = 0.044). *Clostridiaceae* was the second largest family identified in the low %G+C fraction of the cecal microbiome (Figure 5) from the DFM treatment (12.0%). In comparison, the proportion of *Clostridiaceae* family members identified in the control (0.5%) and Narasin (0.2%) treatment groups was significantly lower (*P* = 0.012) and did not comprise a major population in the cecal bacteria identified in these groups. In the Narasin treatment, the low fraction was comprised of a relatively high number of *Erysipelotrichaceae* family members (12.6%), which was higher (*P* = 0.012) than either the control (3.0%) or DFM treatments (1.5%).

## DISCUSSION

Necrotic enteritis is a global issue in poultry production leading to severe economic losses due to reduced growth performance, increased mortality, veterinary associated costs, and poor flock uniformity (Skinner et al., 2010; Timbermont et al., 2011). The causative agent, *C. perfringens*, is a normal inhabitant of the chicken intestinal microbiome, but becomes disease causing when conditions are favorable for proliferation of the species and subsequent population density triggers the expression of toxins (Timbermont et al., 2011; Prescott et al., 2016). The disease most often occurs shortly after the switch from starter to grower feeds in poultry houses, which may be due to disturbances in the intestinal microbiome caused by the adaptation to the new feed (Timbermont et al., 2011). As *C. perfringens* is lacking some amino acid biosynthesis pathways (Shimizu et al., 2002), high protein environments are also positively correlated with NE. For example, *C. perfringens* induced NE is associated with dietary fishmeal inclusion, which increases protein availability to the intestinal microbiome, and infections with parasitic protozoan *Eimeria* spp. that damage the intestinal epithelia and release host plasma proteins into the gut lumen (Timbermont et al., 2011; Antonissen et al., 2016; Prescott et al., 2016). The presence of mycotoxins in the diet and certain vaccine programs have also been associated with increased risk or severity of NE, likely due to immune suppression (McReynolds et al., 2004; Antonissen et al., 2014; Antonissen et al., 2015). Many predisposing factors associated with NE, such as dietary inclusion of fishmeal and *Eimeria* spp. infections, are also thought to disrupt the microbiome resulting in favorable conditions for the outgrowth of *C. perfringens* (Wu et al., 2014).

Probiotics, or DFMs, are proposed as an alternative to prophylactic in-feed antibiotics for the control of NE due to several potential modes of action attributed to DFMs. These mechanisms may include host immune modulation, competition with pathogens for nutrients, direct inhibition of pathogens through metabolite expression, and microbiome stabilization among others (Ducatelle et al., 2015; Elshaghabee et al., 2017). The *B. subtilis* strain DSM 32315 investigated in the present study has been observed to inhibit the growth of *C. perfringens* in vitro (unpublished data). It was, therefore, hypothesized that the strain may be effective for preventing NE by inhibiting the proliferation of *C. perfringens* in the intestines of broiler chicken. The study confirmed that the addition of *B. subtilis* DSM 32315 to the diets of broilers from the starter phase was able to reduce *C. perfringens* populations in the cecum prior to challenge (d 11), as well as the proliferation of *C. perfringens* in the ileum only 2 d after a direct oral inoculation with a virulent strain of the pathogen (d 18), an effect that persisted until the finisher phase (d 35). The reduction of *C. perfringens* in ileum at post-infection time points was additionally observed in the coccidiostat treated birds. It is probable that the inhibition of *C. perfringens* in the ileum was at least partly responsible for the improvements in feed conversion and mortality observed in both the *B. subtilis* DSM 32315 and the Narasin treatments compared to the control. The reduction in footpad lesions in both treatment groups compared to the control is also likely associated with the reduction in *C. perfringens* in the treatments, as footpad lesions have been associated with wet litter conditions, which are often observed in NE (Timbermont et al., 2011; Taira et al., 2014).

While both treatments were effective in controlling *C. perfringens* and ameliorating NE associated detriments to growth performance, the Narasin treatment was superior in the reduction of FCR and intestinal *C. perfringens* populations at some time points. The effectiveness of Narasin is in line with the literature as strains of both *Eimeria* spp. (Ruff et al., 1979; Jeffers, 1981; Jeffers et al., 1988; Smith and Strout, 1980; Weppelman et al., 1977) and *C. perfringens* (Elwinger et al., 1992; Watkins et al., 1997; Elwinger et al., 1998; Martel et al., 2004; Bafundo et al., 2008; Silva et al., 2009) have been observed to be susceptible to the ionophore. However, numerous studies also report Narasin resistance in field strains of *Eimeria* spp. (Bedrnik et al., 1989; Chapman and Hacker, 1994; Djemai et al., 2016; Stallbaumer and Daisy, 1988). Additionally, the presence of toxin gene carrying *C. perfringens* strains was observed in commercial flocks fed diets containing Narasin, suggesting it is not fully effective in preventing the virulence of *C. perfringens* (Engstrom et al., 2012). In regions or production systems where ionophores like Narasin are banned, or in houses where these antimicrobials are observed to be less effective, *B. subtilis* DSM 32315 may offer at least a partial replacement for the control of NE. Combining other products with Narasin for the control of NE has also previously been investigated, often with additive effects compared to dietary treatment of Narasin alone (Bafundo et al., 1989; Brennan et al., 2003). Therefore, a future research avenue may involve investigating additive effects of *B. subtilis* DSM 32315 with various anticoccidials like Narasin for improved protection from NE in shuttling programs in order to reduce *Eimeria* spp. and *C. perfringens* resistance, while targeting both these underlying parasitic and causative bacterial components of NE, respectively.

The addition of probiotics to the diet of broilers has been proposed to lead to the preferential colonization of broilers intestines with beneficial bacterial populations resulting in the establishment of a balanced intestinal microbiome that can prevent opportunistic pathogen populations from proliferating and causing disease (Ducatelle et al., 2015; Elshaghabee et al., 2017). The effects of *B. subtilis* DSM 32315 on the microbiome in the digestive tract of broilers were, therefore, investigated in order to further investigate potential modes of action related to other beneficial bacterial populations supported by the addition of the probiotic in feed. The cecum microbiome was specifically chosen for analysis as it can be a strong indicator of dysbiosis in the small intestine that leads to poor digestion and members of the cecal microbiome have previously been correlated with feed conversion in broilers (Antonissen et al., 2015; Apajalahti and Vienola, 2016; Torok et al., 2011; Stanley et al., 2012; Stanley et al., 2016). The %G+C profiling method of microbiome analysis was utilized as it does not require prior knowledge of the bacterial populations present and is not susceptible to inherent biases of PCR methods (Holben et al., 2004). When the relative abundance of bacterial chromosomes with different %G+C content from the fractionated samples was compared, there were three main regions of interest where the curves were affected by dietary treatment. Sequencing of the 16S rRNA genes of the bacterial chromosomes represented in those fractions identified several family and species differences between the treatment groups in the cecal microbiome of the animals at the peak of NE challenge. This is in contrast to a recent study reporting no effect of dietary inclusion of a *B. subtilis* strain on the cecal microbiome when using 16S sequencing of the microbial genome (Goodarzi Boroojeni et al., 2018). This difference in findings may be due to the study being conducted without an NE challenge or the different *B. subtilis* strain utilized; however, the difference in methodology may also explain the contrasting results. One of the main genus differences observed in our studies was in *Lactobacillus* populations and indeed another recent study comparing the microbial communities found in various intestinal sections of broilers using 16S sequencing of microbial genome reported that *Lactobacillus* were rarely identified in the cecum while 40% of sequences were from the *Bacteroides* genus (Xiao et al., 2017). Perhaps the lack of selection with %G+C profiling prior to sequencing in these studies resulted in an overshadowing of less represented genera and the differences between the species within, by the sequences amplified from the dominant genera.

In the mid-%G+C fraction family distribution, *Lachnospiraceae* was the most highly represented family in all the cecal microbiome of all treatments; however, the DFM treatment significantly decreased this population compared to the negative control which was not observed in the Narasin treatment. The genus level classification revealed that the great majority of *Lachnopiraceae* sequences obtained from this fraction affiliated with a previously isolated strain from cecum of broiler chickens (Bjerrum et al., 2006). This isolate shows the closest homology to *R. torques*, which is known to degrade GI mucin (Wilson et al., 1997). Therefore, the reduction of this population in the DFM fed birds gut microbiome may have improved intestinal mucus structure and be one reason why these animals were more resistant to the NE challenge and had improved performance compared to the controls.

Both DFM and Narasin significantly reduced *Ruminococcaceae* family abundance. *Ruminococcaceae* has been previously reported to be increased in *Eimeria* spp. infections (Wu et al., 2014), so a reduction in this population may have been correlated with the amelioration of the NE challenge model that included a predisposing inoculation of *Eimeria* spp. When species level identification of the sequences obtained from this fraction were investigated, there were significantly fewer *R. lactaris* associated sequences observed in the DFM and Narasin treatment groups compared to the control. While there is little known regarding the effects of this species on broiler health or performance, related strains in the intestinal microbiome of humans have been associated with of irritable bowel syndrome (Hynonen et al., 2016). Additionally, *Ruminococcus* spp. as a group has been correlated with inflammatory cytokine expression in broilers (Oakley and Kogut, 2016). Therefore, it can be hypothesized that a reduction in the presence of *Ruminococcaceae* and specifically *R. lactaris* in the cecal microbiome of broilers in the DFM and Narasin treatment groups could have reduced inflammation and therefore improved gut health in these animals.

The DFM treatment also resulted in significantly greater numbers of *Lactobacillaceae* members compared to both the Narasin treatment and control. The *Lactobacillaceae* species *L. johnsonii* and *L. saliviarius* were specifically increased in the DFM treatment, both of which have been extensively researched for their probiotic properties. *Lactobacillus johnsonii* was previously found in lower numbers in NE infected birds (Stanley et al., 2012) and reported to directly inhibit a NE model when broilers were inoculated with a media of an *L. johnsonii* strain prior to the challenge (Qing et al., 2017). The species was also observed to inhibit the growth of not only *C. perfringens*, but also *Salmonella* spp. and *Escherichia* spp. in vitro (La Ragione et al., 2004). Likewise, *L. salivarius* has been reported to inhibit several foodborne pathogens including various *Salmonella* spp. (Noujaim et al., 2008; Penha Filho et al., 2015; Sornplang et al., 2015; Feng et al., 2016) and *Campylobacter jejuni* (Ghareeb et al., 2012; Saint-Cyr et al., 2017). The pathogen inhibition displayed by *L. salivarius* may be due to bacteriocin production that was previously reported within this species (Svetoch et al., 2011; Saint-Cyr et al., 2017). Other reported probiotic characteristics of *L. salivarius* strains include immune modulation (Noujaim et al., 2008; Penha Filho et al., 2015; Sornplang et al., 2015) and the production of short chain fatty acids like lactic acid (Meimandipour et al., 2009).

It should be noted that there have been confounding reports regarding whether the presence of *Lactobacillaceae* populations in the GI tract of broilers provides a benefit or a detriment to bird health. It has been reported that *Lactobacillaceae* in cecum can be a sign of poor absorption in the upper GI tract (Apajalahti and Vienola, 2016). Additionally, *L. salivarius*, most often reported for having probiotic qualities, was reported to be correlated to poor growth performance of broilers when found in ileum microbiome, possibly due to the potential of some strains to deconjugate bile acids (Torok et al., 2011). However, a negative correlation between a bacterial taxon and performance does not necessarily indicate causality. The divergence in reported associations of intestinal *Lactobacillaceae* populations and broiler health may also be due to differences in bird strain between studies. Difference in the host–microbe interactions between different broiler genetic lines could offer one possible explanation for the confounding reports regarding the correlation of intestinal *Lactobacillaceae* populations with broiler health. A single study investigating the effect of broiler genetic reported that *Lactobacillaceae* populations increased in the ileum of Cobb birds, but decreased in Ross and Hubbard birds during an NE challenge (Kim et al., 2015). This study perhaps offers an alignment between the study reporting that *L. salivarius* in the ileum of Cobb broilers was negatively correlated with growth performance (Torok et al., 2011) and the present study where the DFM fed Ross broilers had higher cecal populations of *Lactobacillaceae*, more specifically *L. salivarius*, and better performance throughout the NE challenge than the controls. The mechanisms behind interaction effects specifically with *Lactobacillaceae* populations and broiler breed are not clear, but could be due to differences in host cell recognition of specific bacterial populations and whether or not various host cell response mechanisms are activated (Abasht et al., 2009; Redmond et al., 2009; Karnati et al., 2015).

The *Clostridiaceae* family was also significantly increased in the DFM treatment group compared to the control or Narasin treatment groups; however, species level analysis showed no significant differences in the abundance of any *Clostridiaceae* species, including *C. perfringens*. The qPCR quantification of *C. perfringens* in the digesta from these same animals also showed no significant decrease in *C. perfringens* populations in the cecum between the treatments. The qPCR did, however, reveal that the DFM treatment did reduce alpha toxin producing *C. perfringens* in the ileum compared to the controls, which is the site of NE infection (Timbermont et al., 2011). The increase in *Clostridiaceae* in the sequenced %G+C fraction of the cecal microbiome of birds fed the DFM compared to the controls is, therefore, not due to any individual species identified, including *C. perfringens*.

The Narasin treatment was not shown to affect the *Lactobacillaceae* or *Clostridiaceae* families in the cecal microbiome of broilers compared to the control; however, Narasin did increase the abundance of *Erysipelotrichaceae* members. The role this family of bacteria may play in gut health is unclear as taxa within this family have been found to induce intestinal inflammation in humans, but also found to be enriched in remission patients compared to active inflammatory bowel disease sufferers (Kaakoush, 2015). In broilers, the findings are also confounding, with one group reporting increased abundance of cecal *Erysipelotrichaceae* in broilers with better feed conversion (Stanley et al., 2016) and another showing an increase in the relative abundance of potentially pathogenic *Erysipelotrichaceae* in the cecum of birds infected with avian leukosis virus compared to uninfected controls (Ma et al., 2017). As no significant differences were observed between treatments with a species level clustering of the sequences, it is difficult to ascertain the potential implications of an increase in this bacterial taxa in cecum of birds from the Narasin treatment group may have had on bird health. Overall, the effects of *B. subtilis* DSM 32315 on cecal microbiome in broilers during a NE challenge were more easily defined on species levels than the effects of Narasin.

In conclusion, the study supports the hypothesis that the addition of *B. subtilis* DSM 32315 spores in broiler feed can control the proliferation of *C. perfringens* in the intestines of broilers in a challenge, prevent performance losses associated with NE, and at least partially replace an in-feed antimicrobial. Further studies to investigate the potential compatibility and synergistic effects of combination treatment with *B. subtilis* DSM 32315 and antimicrobials could provide improved strategies for prevention of NE. Additionally, the DFM was able to support bacterial populations in the intestines with proposed health benefits for the birds while reducing potentially harmful populations. This indicates that supplementation of *B. subtilis* DSM 32315 in broiler feed may stabilize the microbiome and prevent other intestinal disorders resulting from opportunistic pathogens like *Campylobacter jejuni, Escherichia coli*, and various *Salmonella* spp. Finally, the potential effects of feeding the *B. subtilis* DSM 32315 strain on physiological development and function of the GI tract and immune system should be investigated to elucidate other potential mechanisms of action the probiotic may have in supporting the health and growth performance of poultry.
